# The beneficial effect of prophylactic hydrocortisone treatment in extremely preterm infants improves upon adjustment of the baseline characteristics

**DOI:** 10.1038/s41390-023-02785-x

**Published:** 2023-08-31

**Authors:** Olivier Baud, Philippe Lehert, for the PREMILOC study group

**Affiliations:** 1 Division of Neonatology and Pediatric Intensive Care, Children’s University Hospital of Geneva and University of Geneva, Geneva, Switzerland; 2 Faculty of Medicine, University of Melbourne, Melbourne, Australia; 3 Faculty of Economics, University of Louvain, Louvain, Belgium

## Abstract

**Background:**

Prophylactic low-dose hydrocortisone (HC) was found to improve survival without bronchopulmonary dysplasia (BPD) in extremely preterm infants. However, appropriately adjusting for baseline risks of BPD or death might substantially increase the precision of the HC effect size.

**Methods:**

We conducted a secondary analysis of the PREMILOC trial. The treatment effect was evaluated on the primary endpoint through a covariance analysis ANCOVA, adjusting for the baseline covariates using a mixed linear model. Several sensitivity analyses were conducted to assess the potential heterogeneity of the treatment effect across centers and subpopulations.

**Results:**

The interaction between treatment group and baseline risk for BPD or death was not statistically significant (*p* = 0.498). After adjusting for the patient’s probability of BPD-free survival using baseline predictors alone, the HC treatment exhibited a highly significant effect (OR [95% CI] = 2.053 [1.602–2.501], *p* = 0.002), with a number needed to treat NNT [95% CI] = 5.8 [4.1–23.0]. Despite a weak interaction with sex, we found a lack of heterogeneity in the treatment effect across specific subpopulations.

**Conclusions:**

In the PREMILOC trial, the beneficial effect of prophylactic HC versus placebo on BPD-free survival in extremely preterm neonates was found to be greater when adjusted to baseline risks of BPD or death.

**Registration numbers:**

EudraCT number 2007-002041-20, ClinicalTrial.gov number NCT00623740.

**Impact:**

- Prophylactic low-dose hydrocortisone (HC) provided past evidence of a beneficial effect in improving survival without BPD in infants born extremely preterm.
- Adjustment for baseline risks of BPD or death might substantially increase the precision of the HC effect size.
- The beneficial effect of prophylactic HC vs placebo on BPD-free survival in extremely preterm neonates was found to be greater when adjusted to baseline risks of BPD or death.
- We evidenced a lack of heterogeneity in the treatment effect in specific subpopulations despite some weak interaction with sex.

## Introduction

Bronchopulmonary dysplasia (BPD) is a major complication of extreme prematurity with few treatment options.^1,2^ The use of postnatal steroids remains a topic of controversy,^3^ however, prophylactic low-dose hydrocortisone (HC) has emerged as a promising intervention in preventing functional adrenal insufficiency soon after birth in extremely preterm infants, while also mitigating the adverse impact of inflammation on lung development.^4^ Moreover, HC has demonstrated potential in improving survival rates without BPD.^4^

The PREMILOC trial reported a statistically significant reduction in BPD-free survival in extremely preterm infants treated with HC at birth (60% in the HC group versus 51% in the placebo group (OR [95% CI] = 1.48 [1.02–2.16], *p* = 0.04; NNT [95% CI] = 12 [6–200]),^5^ notably without long-term neurodevelopmental adverse outcomes.^6–8^ A recent meta-analysis, utilizing individual patient data, provided further confirmation regarding the beneficial effects of HC. The analysis revealed significant improvements not only in survival rates without BPD (OR [95% CI] = 1.45 [1.11–1.90], *p* = 0.007; *I*^2^ = 0%), but also in the medical treatment for patent ductus arteriosus (OR [95% CI] = 0.72 [0.56–0.93], *p* = 0.01; *I*^2^ = 0%), and a reduced risk of death before discharge (OR [95% CI] = 0.70 [0.51–0.97], *p* = 0.03; *I*^2^ = 0%).^9^

The PREMILOC trial was conducted under a group sequential design which involved interim analyses for ethical reasons. The trial was stopped by the steering committee, leading to the analysis of the primary endpoint on a smaller sample size than originally planned. Despite the limited sample size of 521 infants compared to the intended 786 subjects, a significant benefit of HC was observed. However, due to the constraints of the sequential design, the inferential tool used was limited to an unadjusted *χ*^2^ test for comparing proportions between two independent samples. This double limitation of a reduced sample size and an unadjusted inferential test resulted in a trial that lacked sufficient power, leading to less precise estimates and the inability to investigate the homogeneity of the HC effect across different baseline conditions. Considering the importance of HC as a treatment for BPD in neonates, it is crucial to conduct a comprehensive statistical re-analysis to strengthen the evidence. This re-analysis aims to improve power, provide a more accurate estimate of the effect size, and reduce the overall residual variability of the endpoints by considering baseline covariates known to significantly impact the outcomes, as previously demonstrated in the NICHD vitamin A trial.^10,11^

## Patients and methods

### Study population and database

This was an exploratory re-analysis of the PREMILOC trial, which was a multicenter, double-blinded, placebo-controlled randomized trial. The trial involved the enrollment of 523 extremely preterm infants born before 28 weeks of gestation from 21 perinatal centers in France between 2008 and 2014. Infants meeting the eligibility criteria were randomly assigned to receive either prophylactic low-dose HC or placebo within the first 10 days after birth. All enrolled infants were inborn, delivered before 27 completed weeks of gestation, and included within 24 h after birth. The primary outcome assessed was survival without BPD at 36 weeks postmenstrual age (PMA), and the analysis was performed on a subset of 521 infants. The trial was approved by the French national ethics committee (Comité de Protection des Personnes (CPP), Ile-de-France II, Necker), the French National Drug Safety Agency (ANSM, EudraCT number 2007-002041-20), and the French data protection authority (Commission Nationale de l’Informatique et des Libertés; CNIL). Prior to randomization, written informed consent was obtained from the parents of all eligible infants. The trial was registered on ClinicalTrials.gov (NCT00623740) prior to enrolling the first patient. The study protocol has been previously reported. In summary, the infants in the trial received either a placebo or HC treatment. The HC dosage regimen involved administering 1 mg/kg per day, divided into two equal doses, for a duration of seven days. This was followed by a reduced dosage of 0.5 mg/kg per day for three additional days. The specific HC used was UPJOHN 100 mg for injection, manufactured by SERB Laboratories in Paris, France.

### Statistical methods

The primary aim of this study was to conduct a reassessment of the PREMILOC trial using a statistical model that would enhance the precision of the HC effect size and evaluate the consistency of the treatment effect across various baseline subgroups defined by baseline covariates. To ensure rigor and transparency, a comprehensive statistical plan was established and agreed upon prior to any data processing. This plan consisted of two distinct steps.

In the first step, we developed a predictive model, independent of treatment considerations, by conducting a systematic review of risk factors associated with BPD. This model served as an external validation and improvement of the NICHD BPD estimator’s discriminatory ability. The baseline predictors included gestational age at birth, birth weight, respiratory support at baseline (RSB), sex, center effect, and multiple pregnancy status. The severity of RSB was categorized into three groups: mild (non-invasive ventilation with FiO_2_ < 30%), moderate (invasive mechanical ventilation with FiO_2_ < 30%), and severe (invasive mechanical ventilation with FiO_2_ ≥ 30%). Our newly developed model exhibited appropriate calibration and successfully classified 81% of patients.^12^

In the second step, we conducted a reassessment of the effect of HC compared to placebo using our predictive model. The primary outcome of interest was the occurrence of survival without BPD at 36 weeks PMA. To evaluate the significance of the HC effect on this endpoint, we employed a covariance analysis ANCOVA, adjusting for the baseline covariates included in our predictive model. We initially assumed the homogeneity of the treatment effect across the baseline covariates. The analysis was carried out using a mixed linear model, which allowed us to incorporate both fixed and random covariates. We included the center (study site) as a random factor in the model to assess the significance of the standard deviation of the primary endpoint across the sites involved in the study. Importantly, our analysis strictly adhered to the intent-to-treat principle, ensuring the selection of participants based on their original treatment assignment without any exclusions. Our main findings were subject to several sensitivity analyses to explore different aspects of the treatment effect:

- We conducted an analysis to assess the potential heterogeneity of the treatment effect across different centers. This involved incorporating a random effect for treatment across centers in addition to the intercept in our model.
- To account for the fact that infants who died before 3 postnatal days may be marginally eligible for HC treatment (as the clinical effect requires a 10-day duration), we performed an analysis excluding these infants and re-evaluated the HC effect using the same model.
- While our main model assumed the homogeneity of the HC treatment effect across baseline covariates, we conducted additional tests to explore the presence of subgroups defined by baseline conditions. For each baseline covariate, we introduced an interaction term between the treatment effect and that specific covariate. We then tested the significance of these interaction terms at a significance level of *p* = 0.05.
- Furthermore, we incorporated the prediction model developed in the previous section, which allowed us to assign each patient a probability of success (survival without BPD) or an expected level of therapy response. This prediction was included as a covariate in the main model, enabling us to assess the homogeneity of the HC effect using a similar interaction test.

By conducting these sensitivity analyses, we aimed to comprehensively explore different aspects of the HC treatment effect and investigate potential sources of heterogeneity or interaction effects. To assess the presence of interaction effects, we compared our main model to alternative models by evaluating the significance (*p* < 0.05) of changes in the residual sum of squares (RSS) and the improvement (reduction) of the Bayesian Information Criterion (BIC), which measures the level of information. These comparisons were conducted to identify any significant improvements in model fit. Additionally, to provide a descriptive overview, we complemented our model with a two-way mean table adjusted for each important baseline covariate.

To visually depict the effect of the treatment while considering the simultaneous variation of baseline predictors, the final model was represented by its marginal estimates.

In all tables, percentages were rounded to one decimal place, *p* values were rounded to three decimals, and *p* values less than 0.001 were reported as <0.001. All statistical tests were performed at a two-sided significance level of 0.05. The statistical analyses were carried out using R release 4.1.0 for Windows (R Core team, 2021).

## Results

Out of the initially enrolled 523 extremely preterm infants, 256 were allocated to the placebo group, while 267 were assigned to the HC group. For the analysis conducted, a total of 519 infants were included, as consent was withdrawn by the parents of one child in the HC group and three children in the placebo group. Detailed prenatal, baseline, and respiratory characteristics before treatment allocation can be found in Supplementary Tables 1–3. Importantly, no significant differences were observed between the two groups for any of the reported variables.

The primary endpoint of interest was survival without BPD at 36 weeks of PMA. Among the placebo group, 135 out of 264 infants (51.1%) achieved this outcome, whereas in the HC treatment group, 153 out of 255 infants (60.0%) achieved the same outcome. The comparison using a *χ*^2^ test showed a significant difference between the two groups (*p* = 0.04). Our primary analysis utilized a mixed logistic regression model, which was adjusted for the baseline predictors of our predictive model as shown in Table 1. Among the most prevalent categories of baseline conditions, our reference population consisted of male infants weighing 850 g at birth and delivered at 26 weeks gestation, with mild respiratory support at baseline.

**Table 1:** Logistic mixed model regression.

	Effect (OR)	Se	95% CI	*p* value	Center
Overall estimate	63.6%	28.9%	49.8–75.5%	<0.001	0.472
Gestational age	1.504	0.147	1.127–2.006	0.006	–
Birth weight	1.006	0.001	1.004–1.008	<0.001	–
Female sex	2.843	0.244	1.762–4.588	<0.001	–
RSB—moderate	0.389	0.272	0.228–0.664	0.001	–
RSB—severe	0.099	0.381	0.047–0.208	<0.001	–
Multiple pregnancy	0.475	0.245	0.294–0.767	0.002	–
HC treatment	1.820	0.229	1.162–2.852	0.009	–

The dependent variable is BPD-free survival. The first row (overall estimate) provides the estimated proportion of the endpoint for the reference population, and the relative variation of this value across centers. The following rows report the effect of each covariate in the model by its odds ratio (OR), standard error (Se), associated 95% confidence interval (CI) and *p* values. Last column report the relative error (variation coefficient) of the studied endpoint across categories of each random factor.

For this reference population, the estimated proportion of BPD-free survival rate was found to be 63.6% (95% CI: 49.8–75.5%), with a notable relative variation observed across centers (coefficient of variation (CV) = 0.48).

After adjusting for the covariates included in the baseline model and comparing it to the control group, a beneficial effect of HC treatment was identified. The odds ratio (OR) was determined to be 1.82 [95% CI: 1.16–2.85], with a *p* value of 0.009. This effect size can be interpreted as a risk ratio (RR) of 1.30 [1.07–1.48] or equivalently as an absolute risk difference (ARD) of 14.6% [0.04–24.3]. The derived number needed to treat (NNT) was calculated as 6.9 [4.2–27.0], indicating the number of patients who would need to be treated to observe one additional favorable outcome.

**Fig. 1:**
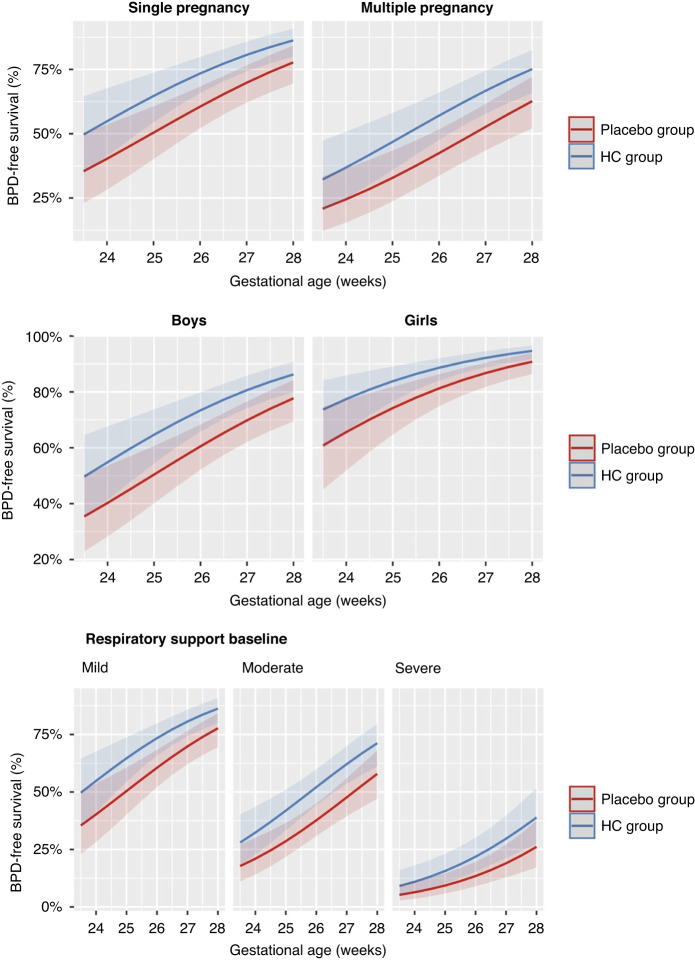
Probability of BPD-free survival according to baseline predictors of BPD/death and gestational age. Each graphic represents data for pregnancy category, sex, and respiratory support severity at baseline. In each graphic, the difference between the two treatment groups, Placebo and HC, is provided with the prediction confidence intervals at 90% significance.

Figure 1 graphically presented the revised model, depicting the estimated marginal values of BPD-free survival across different factors, including gestational age at birth, respiratory support at baseline (RSB) severity, sex, and pregnancy category. We evaluated the metric qualities of the resulting predictive model when incorporating the HC treatment. The determination coefficient (*R*^2^) for this model was determined to be 0.492. Additionally, compared to our original model, the addition of the treatment led to a slight increase in the area under the receiver operating characteristic curve (AUC-ROC) to *C* = 0.86 [0.82 to 0.89]. Furthermore, the calibration of the model was confirmed through the Hosmer–Lemeshow test and the proportionality between the expected and observed frequencies, with the intercept estimated at 0.002 [−0.046 to 0.050] and the slope at 1.005 [0.929 to 1.080]. Supplementary Fig. 1 provides a visual representation of these findings.

Sensitivity analyses were conducted to assess the consistency of the main results obtained. The following analyses were performed:

1. Heterogeneity across centers: we examined the possibility of a treatment effect heterogeneity across different centers. The model used showed negligible random factor variation (CV = 0.094) for the treatment efficacy across centers, as presented in Supplementary Table 4.
2. Exclusion of early deaths: to account for the likelihood that very early deaths (within 3 days after birth) may be too premature for treatment, we repeated the analysis after excluding these cases. The results remained consistent, with a slightly increased odds ratio (OR [95% CI] = 1.833 [1.17–2.87], *p* = 0.008), as shown in Supplementary Table 5 (sample size: 511).
3. Assessment of treatment effect homogeneity across subgroups: we evaluated the homogeneity of the treatment effect across subgroups defined by baseline conditions included in the predictive model. No significant interactions were observed between treatment and gestational age at birth effect, (OR [95% CI] = 1.024 [0.626–1.675, *p* = 0.922, Supplementary Table 6), birth weight (OR [95% CI] = 0.998 [0.994–1.002, *p* = 0.143, Supplementary Table 7), multiple pregnancy (OR [95% CI] = 0.573 [0.231–1.420], *p* = 0.229, Supplementary Table 8), or between RSB severity (Supplementary Table 9). However, a significant interaction was found between sex and treatment effect (OR [95% CI] = 2.651 [1.078–6.520], *p* = 0.034). Notably, the Bayesian Information Criterion (BIC) associated with this interaction was not lower than the BIC of the main model, as shown in Supplementary Table 10. Overall, no statistically significant interactions between treatment effect and baseline covariates were detected.
4. Homogeneity across the predicted probability of BPD-free survival: our predictive model provided the patient’s probability of BPD-free survival based solely on baseline predictors available at or soon after birth. We examined the homogeneity of the HC treatment effect across the range of this probability, as presented in Table 2. Despite the statistically significant effect of the predictive model (OR [95% CI] = 1.058 [1.046–1.063], *p* < 0.001), the adjusted treatment effect remained highly significant (OR [95% CI] = 2.053 [1.602–2.501], *p* = 0.002). This corresponded to a RR of 1.34 [1.09–1.49], an absolute risk difference (ARD) of 17.2% [0.05–25.8], or a number needed to treat (NNT) of 5.8 [4.1–23.0]. No significant interaction effect was detected between treatment and the baseline probability of BPD-free survival (OR [95% CI] = 0.990 [0.976–1.012], *p* = 0.498). However, Supplementary Fig. 2 displayed two curves representing the probability of therapy response for placebo (red) and HC (blue), showing a somewhat larger effect for patients with a likelihood of BPD-free survival below 75%. Conversely, for patients with a favorable prognosis, the two curves tended to overlap. Due to the sample size limitations, this effect could not be quantified precisely. Nevertheless, Table 3 provides unadjusted success proportions estimated in four groups defined by 25%, 50%, and 75% cut points of the prediction, illustrating this finding.

**Table 2:** Logistic mixed model regression assessing the estimated effect of the predicting probability (Predict) of success (BPD-free survival) at baseline for patients without treatment, HC treatment, and interaction effect of Predict with treatment (Predict:trt).

	Effect (OR)	Se	95% CI	*p* value
Intercept	0.510	0.168	0.182–0.839	0.031
Predict	1.058	0.007	1.045–1.071	<0.001
HC treatment	2.053	0.230	1.602–2.505	0.002
Predict:trt	0.994	0.009	0.976–1.012	0.498

**Table 3:** Success proportions between treatment groups assessed for each category of patients classified according to their probability to survive without BPD at baseline.

Prognosis at baseline	Placebo	Hydrocortisone	Relative risk (95% CI)
Worst (<25%)	5/53 (9.4%)	10/55 (18.2%)	1.55 (0.84–3.47)
Fair (25–50%)	13/51 (25.5%)	26/58 (44.8%)	1.63 (1.03–2.74)
Good (50–75%)	36/62 (58.1%)	38/54 (70.4%)	1.27 (0.90–1.77)
Best (>75%)	81/98 (82.7%)	79/88 (89.8%)	1.29 (0.89–1.70)
Overall population	135/264 (51.1%)	153/255 (60.0%)	1.19 (1.01–1.41)

## Discussion

Previous evidence has indicated a positive effect of prophylactic low-dose HC in improving survival rates without BPD among extremely preterm infants. However, to enhance the precision of the effect size and gain a deeper understanding of the impact of baseline conditions and specific subgroups, further investigation was required. Thus, the primary objective of this study was to address these gaps in knowledge and provide a more comprehensive assessment of the HC treatment in this population. Our initial objective was to provide a more precise estimate of the effect of HC treatment. The original analysis, which employed a simple unadjusted test, reported a significant increase in survival without BPD among neonates exposed to HC, with an OR of 1.48, equivalent to a RR of 1.19 [1.01–1.38]. In our re-analysis, adjusting for baseline predictors, we observed an OR of 1.82, corresponding to a RR of 1.30 [1.07–1.48], and a number NNT of 6.9 [4.2–27.0]. This finding aligns with our expectations, as it takes into account the reduced residual variability accounted for by fixed covariates, along with the supplementary effects of the centers. These factors, coupled with a high determination of the model (*R*^2^ = 0.505), contribute to the improved precision of our estimate.

While the original analysis had a power of 0.59, considering a difference of 10%, the increased determination of the model enhances the power to 0.89. This demonstrates the increased statistical robustness of our re-analysis compared to the original PREMILOC results. The clinical relevance of a drug’s effect is often best understood when expressed in terms of the RR. In our sensitivity analysis, we obtained a range of RR values between 1.30 and 1.34. From a statistical perspective, given our reference population’s responder proportion of 63.6%, the maximum attainable RR would be 1.57. Therefore, the observed efficacy of HC reaches 52.6% of the maximum potential efficacy.

Additionally, a RR of at least 1.30 can be translated into a Cohen’s^13^ *d* difference of 0.41, which is considered close to a medium clinically relevant effect (*d* = 0.5). From a clinical standpoint, it is worth noting that HC, based on the available evidence, currently represents the most well-supported treatment option. Furthermore, our intent-to-treat analysis considers the net responder rate, taking into account both survival and death outcomes.

Considering these various arguments, we can conclude that our findings provide some evidence of a clinically relevant difference associated with HC treatment.

An interesting observation in our study was the notable variation (CV = 0.47) among different centers, indicating a significant center effect on the primary endpoint. Recognizing the importance of this center effect and its potential implications, we conducted a sensitivity analysis to examine the possible heterogeneity of the treatment effect across centers.

In this analysis, we evaluated the random effect of the treatment across centers and found that the variation across centers was small and non-significant (0.093, Supplementary Table 11). It is worth noting that this aspect is often overlooked, despite its relevance. Exploring potential heterogeneity in the treatment effect can help identify specific subpopulations that may experience greater benefit or harm.^14^ A notable example of such analysis was previously reported by Doyle et al., who conducted a meta-regression to assess the benefit–risk ratio of using postnatal steroids for BPD while considering the risk of neurodevelopmental impairment.^15^

Our findings indicate that although the success proportion of BPD-free survival varies significantly among the different participating centers, the effect of the treatment remains consistent across these centers. This implies that the impact of the drug is relatively constant regardless of the center where the treatment is administered.

It is widely recognized that centers can differ in terms of their patient characteristics, clinical practices, and available resources, which often lead to variations in success proportions.^16^ However, in our study, we observed a homogeneous efficacy of HC across centers, indicating that there was no significant interaction between the center and the effect of HC treatment.

This suggests that the benefits of HC treatment are not influenced by the specific center where the treatment is administered, supporting the generalizability and consistency of its effect across diverse clinical settings.

An unanswered question that remained was the extent to which the HC treatment provides homogeneous benefits across the entire population or if certain subgroups of neonates, defined by their baseline conditions, may exhibit differences in treatment response. To address this question, we conducted an analysis to assess the homogeneity of the treatment effect across subgroups defined by the baseline conditions encompassing all predictors included in our model. This was achieved by testing the interaction term between each predictor and the treatment.

Our findings indicate no apparent interaction between gestational age, weight, pregnancy status, or respiratory support and the HC treatment. This suggests that the beneficial effect of HC is consistent and homogeneous across these baseline conditions. These results allow us to conclude that the HC treatment provides an overall homogeneous benefit, independent of any specific baseline conditions. Among the various baseline conditions evaluated, the only significant interaction observed was in relation to sex. However, it is important to interpret this interaction with caution due to the wide confidence interval. It is worth noting that an individual patient data meta-analysis, which included a substantial number of infants enrolled in randomized controlled trials assessing the effect of low-dose early hydrocortisone, reported similar effects for both males (OR [95% CI] = 1.40 [0.97–2.02]) and females (OR [95% CI] = 1.52 [1.02–2.26]).^9^ This suggests that the potential difference in treatment response based on sex, as observed in the PREMILOC cohort, requires further investigation and confirmation through additional studies or meta-analyses with more robust data. The absence of a difference in our study can be attributed to inconsistent findings regarding the effect of sex in the two main randomized controlled trials that have investigated the impact of low-dose prophylactic HC treatment.^5,17^ In the PREMILOC trial, females were observed to benefit more from HC treatment, while in the PROPHET trial, males appeared to be the better responders to HC. These divergent results from the two trials contribute to the lack of a clear sex effect observed in our study. Further research is needed to better understand the potential sex-specific treatment responses and to elucidate the underlying factors contributing to these discrepancies.

Another analysis was conducted to examine the effect size based on the initial severity of patients by assessing the homogeneity of the effect over the overall baseline variability, as represented by the expected probability of BPD-free survival calculated using our predictive model. The results of this analysis demonstrated that the positive effect of HC treatment remained consistent regardless of the predicted outcome at baseline, although there was a trend indicating a better response in infants with a chance of BPD-free survival below 75%.

To the best of our knowledge, this is the first analysis to report on the heterogeneity of treatment effect in a randomized trial involving corticosteroid exposure in extremely preterm infants. Another strength of our study is the comprehensive baseline prediction of BPD, which covered a wide distribution of risks, enabling us to address this important question.

However, it is important to acknowledge a limitation of our study, which is the lack of assessment of the heterogeneity of treatment effect for safety outcomes such as secondary sepsis, spontaneous intestinal perforation, or neurodevelopmental impairment. Nevertheless, previous findings from the PREMILOC trial have indicated that HC exposure did not have a significant impact on outcomes such as spontaneous intestinal perforation and neurodevelopmental outcomes.^5–7^

In conclusion, this study provides further confirmation that prophylactic low-dose HC offers statistically and clinically significant benefits in improving BPD-free survival in infants born extremely preterm. An encouraging finding of this study is the consistent direction of the treatment effect observed across various baseline conditions, supporting the robustness of the HC treatment in this population.

### Supplementary Information

Appendix

Supplemental materials

## Data Availability

The data supporting the findings of this study are available from Assistance Publique Hôpitaux de Paris; however, access to these data is subject to certain restrictions as they were used under license for the current study. Therefore, the data are not publicly available. Nonetheless, researchers may request access to the data from the authors, subject to approval from Assistance Publique Hôpitaux de Paris.
