# Supplementary material for: The beneficial effect of prophylactic hydrocortisone treatment in extremely preterm infants improves upon adjustment of the baseline characteristics
Source: Pediatr Res. 2023 Aug 31;95(1):251–6. doi: 10.1038/s41390-023-02785-x (PMC10798883; doi:10.1038/s41390-023-02785-x)
Supplement: Supplementary file 2 — Supplemental materials [file 41390_2023_2785_MOESM2_ESM.pdf]

Supplemental materials

Variable	Placebo (n=264)	Hydrocortisone (n=255)
Mother Age (years)<br>Mean ± SD<br>Median [IQR]	 30.3 ± 5.6<br>29.7 [26.6, 34.7]	 30.5 ± 5.6<br>29.9 [26.5, 34.5]
Father ethnic group<br>- Caucasian<br>- African<br>- Asian<br>- Other	 120 (48.8%)<br>83 (33.7%)<br>11 (4.5%)<br>32 (13.0%)	 113 (49.3%)<br>79 (34.5%)<br>6 (2.6%)<br>31 (13.5%)
Mother ethnic group<br>- Caucasian<br>- African<br>- Asian<br>- Other	 111 (43.7%)<br>96 (37.8%)<br>12 (4.7%)<br>35 (13.8%)	 113 (49.3%)<br>79 (34.5%)<br>6 (2.6%)<br>31 (13.5%)
Multiple pregnancy	90 (34.1%)	82 (32.2%)
Epidural analgesia	117/254 (46.1%)	106/241 (44.0%)
General anesthesia	29/255 (11.4%)	40/246 (16.3%)
Tocolytics	181/263 (68.8%)	160/254 (63.0%)
Antenatal antibiotics	184 (69.7%)	173 (67.8%)
Antenatal steroids	244 (92.4%)	238 (93.3%)
Chorioamnionitis	125/237 (52.7%)	113/227 (49.8%)
Premature rupture of membranes > 24h	82 (31.1%)	76 (29.8%)
Gestational diabetes	17/255 (6.7%)	11/250 (4.4%)
Gestational hypertension	23 (8.7%)	34 (13.3%)

Supplementary Table 1:
Prenatal characteristics of infants included in the re-analysis.
SD means standard deviation
IQR means interquartile range

Variable	Placebo (n=264)	Hydrocortisone (n=255)
Cesarean section	123 (46.6%)	121 (47.5%)
Birthweight (g)<br>Mean ± SD<br>Median [IQR]	 862 ± 161<br>848 [735, 970]	 867 ± 151<br>860 [750, 970]
Gestational age (days)<br>Mean ± SD<br>Median [IQR]	 26.3 ± 0.9<br>26.5 [25.7, 27.1]	 26.4 ± 0.9<br>26.4 [25.6, 27.1]
Female sex	116 (43.9%)	124 (48.6%)
Apgar Score at 10mn<br>Mean ± SD<br>Median [IQR]	n=201<br>9.0 ± 1.2<br>9.0 [8.0, 10.0]	n=195<br>8.8 ± 1.5<br>9.0 [8.0, 10.0]
Randomization age (h)<br>Mean ± SD<br>Median [IQR]	 14.7 ± 4.4<br>15.0 [11.0, 18.0]	 15.0 ± 4 .5<br>16.0 [12.0, 18.0]
CRIB score<br>Mean ± SD<br>Median [IQR]	n=205<br>4.5 ± 3.6<br>4.0 [1.0, 7.0]	n=207<br>4.4 ± 3.4<br>4.0 [1.0, 6.0]

<u>Supplementary Table 2</u>:
Characteristics at birth of infants included in the re-analysis.
SD means standard deviation
IQR means interquartile range
CRIB means Clinical Risk Index for Babies

Variable	Placebo (n=264)	Hydrocortisone (n=255)
Ventilatory support<br>- MV<br>- HFO<br>- NIPPV or nCPAP	n=261<br>138 (52.9%)<br>26 (10.0%)<br>97 (37.2%)	n=250<br>129 (51.6%)<br>27 (10.8%)<br>94 (37.6%)
FiO_2 before randomization (%)<br>Mean ± SD<br>Median [IQR]	n=258<br>24.8 ± 8.8<br>21.0 [21.0, 25.5]	n=247<br>24.9 ± 9.7<br>21.0 [21.0, 25.0]
FiO_2 before randomization < 30%	225 (85.2%)	206 (80.8%)
FiO_2 max before randomization (%)<br>Mean ± SD<br>Median [IQR]	n=179<br>47.7 ± 18.6<br>43.0 [35.0, 58.0]	n=185<br>48.3 ± 17.4<br>43.0 [36.0, 59.0]
RSB score<br>Mean ± SD<br>Median [IQR]	 0.78 ± 0.65<br>1.00 [0.00, 1.00]	 0.82 ± 0.71<br>1.00 [0.00, 1.00]
Oxygenation index<br>Mean ± SD<br>Median [IQR]	n=142<br>3.3 ± 2.7<br>3.1 [1.8, 4.4]	n=146<br>3.5 ± 2.9<br>3.6 [1.9, 4.7]
MV MAP (cmH_20)<br>Mean ± SD<br>Median [IQR]	n=164<br>7.4 ± 2.4<br>7.0 [6.0, 7.6]	n=141<br>7.4 ± 2.0<br>7.0 [6.4, 7.7]
HFO MAP (cmH_20)<br>Mean ± SD<br>Median [IQR]	n=13<br>9.8 ± 4.7<br>8.7 [6.8, 12.1]	n=16<br>9.3 ± 2.5<br>9.0 [8.2, 11.5]
NIPPV or nCPAP PEEP (cmH_20)<br>Mean ± SD<br>Median [IQR]	n=44<br>4.8 ± 1.0<br>4.9 [4.0, 5.0]	n=50<br>4.6 ± 0.7<br>4.6 [4.0, 5.0]

Supplementary Table 3:
Characteristics at baseline of ventilatory support of infants included in the re-analysis.
SD means standard deviation
IQR means interquartile range
MV means mechanical ventilation
HFO means high-frequency ventilation
NIPPV means non invasive positive pressure ventilation
nCPAP means nasal continuous positive airway pressure
RSB means respiratory support at baseline; score was calculated as described in the methods section
MAP means mean airway pressure

	Effect (OR)	Se	95% CI	P-value	Center
Overall estimate	0.636	0.283	0.501 to 0.753	0.048	0.421
Gestational age	1.502	0.147	1.126 to 2.004	0.006	-
Birhweight	1.006	0.001	1.004 to 1.008	<0.001	-
Female sex	2.849	0.244	1.766 to 4.597	<0.001	-
RSB-moderate	0.390	0.270	0.230 to 0.663	0.001	-
RSB-severe	0.098	0.381	0.047 to 0.208	<0.001	-
Multiple pregnancy	0.474	0.245	0.293 to 0.767	0.002	-
HC treatment	1.804	0.232	1.145 to 2.843	0.011	0.094

Supplementary Table 4:
Logistic mixed model regression assessing the random effect of the treatment across centers. The dependent variable is BPD-free survival. The first row (overall estimate) provides the estimated proportion of the endpoint for the reference population, and the relative variation of this value across centers. The following rows report the effect of each covariate in the model by its Odds Ratio (OR), standard error (Se), associated 95% confidence interval (CI) and P-values.
Last column report the relative error (variation coefficient) of the studied endpoint across categories of each random factor. Dispersion of the random effect of the treatment across centers was found to be 9.4% only.

	Effect (OR)	Se	95% CI	P-value	Center
Overall Estimate	0.635	0.288	0.497 to 0.754	0.054	0.466
Gestational age	1.490	0.147	1.117 to 1.988	0.007	-
Weight	1.006	0.001	1.004 to 1.008	<0.001	-
Female sex	2.843	0.243	1.766 to 4.579	<0.001	-
RSB-moderate	0.387	0.271	0.227 to 0.658	<0.001	-
RSB-severe	0.113	0.387	0.053 to 0.241	<0.001	-
Multiple Pregnancy	0.481	0.246	0.297 to 0.779	0.003	-
HC treatment	1.833	0.229	1.170 to 2.872	0.008	-

Supplementary Table 5:
Logistic mixed model regression. Treatment effect in eliminating early deaths (less than 3 days after birth).

	Effect (OR)	Se	95% CI	P-value	Center
Overall estimate	0.636	0.289	0.498 to 0.755	0.054	0.472
Gestational age	1.486	0.191	1.022 to 2.161	0.038	-
HC treatment	1.828	0.232	1.160 to 2.880	0.009	-
Birthweight	1.006	0.001	1.004 to 1.008	<0.001	-
Female sex	2.843	0.244	1.762 to 4.588	<0.001	-
RSB-moderate	0.389	0.272	0.228 to 0.663	0.001	-
RSB-severe	0.099	0.381	0.047 to 0.208	<0.001	-
Multiple pregnancy	0.475	0.245	0.294 to 0.768	0.002	-
Gest-Age:trt	1.024	0.251	0.626 to 1.675	0.922	-

Supplementary Table 6:
Logistic mixed model regression assessing the interaction between gestational age at birth and treatment effect on the proportion of infants surviving without BPD. BIC without/with interaction = 559/565.

	Effect (OR)	Se	95% CI	P-value	Center
Overall Estimate	0.636	0.291	0.497 to 0.756	0.055	0.474
Gestational age	1.504	0.148	1.125 to 2.010	0.006	-
Birthweight	1.008	0.001	1.006 to 1.010	<0.001	-
HC Treatment	1.791	0.229	1.143 to 2.807	0.011	-
Female sex	2.832	0.245	1.752 to 4.578	<0.001	-
RSB-moderate	0.390	0.272	0.229 to 0.664	0.001	-
RSB-severe	0.098	0.380	0.047 to 0.207	<0.001	-
Multiple pregnancy	0.473	0.246	0.292 to 0.767	0.002	-
Birthweight:trt	0.998	0.002	0.994 to 1.002	0.143	-

Supplementary Table 7:
Logistic mixed model regression assessing the interaction between birthweight and treatment effect on the proportion of infants surviving without BPD. BIC without/with interaction= 559/563.

	Effect (OR)	Se	95% CI	P-value	Center
Overall Estimate	0.620	0.298	0.476 to 0.745	0.101	0.470
Gestational age	1.502	0.147	1.126 to 2.006	0.006	-
Weight	1.006	0.001	1.004 to 1.008	0.001	-
Female sex	2.807	0.244	1.740 to 4.531	0.001	-
Treatment	2.121	0.282	1.220 to 3.688	0.008	-
Rsb-moderate	0.394	0.272	0.231 to 0.672	0.001	-
Rsb-severe	0.098	0.381	0.046 to 0.207	0.001	-
Multiple Pregnancy	0.591	0.338	0.305 to 1.147	0.120	-
Trt:pregn	0.641	0.474	0.253 to 1.624	0.349	-

Supplementary Table 8:
Logistic mixed model regression assessing the interaction between multiple pregnancy and treatment effect on the proportion of infants surviving without BPD. BIC without/with interaction: 559/565

	Effect (OR)	Se	95% CI	P-value	Center
Overall Estimate	0.673	0.333	0.517 to 0.798	0.030	0.472
Gestational age	1.505	0.147	1.129 to 2.010	0.005	-
Weight	1.006	0.001	1.004 to 1.008	0.001	-
Female sex	2.826	0.244	1.751 to 4.559	0.001	-
Treatment	1.273	0.414	0.565 to 2.863	0.561	-
Rsb-moderate	0.301	0.364	0.147 to 0.616	0.001	-
Rsb-severe	0.085	0.584	0.027 to 0.268	0.001	-
Multiple Pregnancy	0.467	0.247	0.288 to 0.758	0.002	-
Trt:rsb-moderate	1.740	0.514	0.636 to 4.764	0.281	-
Trt:rsb-severe	1.394	0.754	0.318 to 6.110	0.660	-

Supplementary Table 9: Logistic mixed model regression assessing the interaction between RSB severity and treatment effect on the proportion of infants surviving without BPD. BIC without/with interaction = 559/570.

	Effect (OR)	Se	95% CI	P-value	Center
Overall estimate	0.682	0.309	0.540 to 0.798	0.013	0.489
Gestational age	1.507	0.149	1.125 to 2.018	0.006	-
Birthweight	1.006	0.001	1.004 to 1.008	<0.001	-
Female sex	1.793	0.323	0.952 to 3.378	0.070	-
HC treatment	1.182	0.305	0.650 to 2.149	0.585	-
RSB-moderate	0.382	0.273	0.224 to 0.653	<0.001	-
RSB-severe	0.094	0.387	0.044 to 0.201	<0.001	-
Multiple Ppregnancy	0.490	0.248	0.301 to 0.796	0.004	-
Sex:trt	2.651	0.459	1.078 to 6.520	0.034	-

Supplementary Table 10:
Logistic mixed model regression assessing the interaction between sex and treatment effect on the proportion of infants surviving without BPD. BIC without/with interaction = 559/561.

Response % of HC group	ARD	R^2=0	0.25	0.5	0.75
55%	5%	0.211	0.222	0.265	0.414
57.5%	7.5%	0.410	0.432	0.516	0.745
60%	10%	0.640	0.668	0.768	0.939
62.5%	12.5%	0.828	0.852	0.919	0.993
65%	15%	0.939	0.952	0.982	1.000

Supplementary Table 11: Power calculation according to absolute risk difference (ARD) between HC and placebo groups and et the determination coefficient R^2 of the baseline predictive risk model.

In this table, we calculated the power associated with an absolute risk difference ARD varying from 5% until 15% (a range covering the observed ARD of 8.9%), assuming a placebo response proportion of 50%. This calculation is based on a fixed sample of 519 subjects (≅260 subjects/group), the proportion of 50% success rate in the control group, and the use of a two-sided 0.05 test. We provide here the power associated with different values of the Nagelkerke correlation coefficient (NCC in rows), and the expected proportion of success in the studied treatment group (subjects reaching the primary endpoint) (from 55% until 75%).

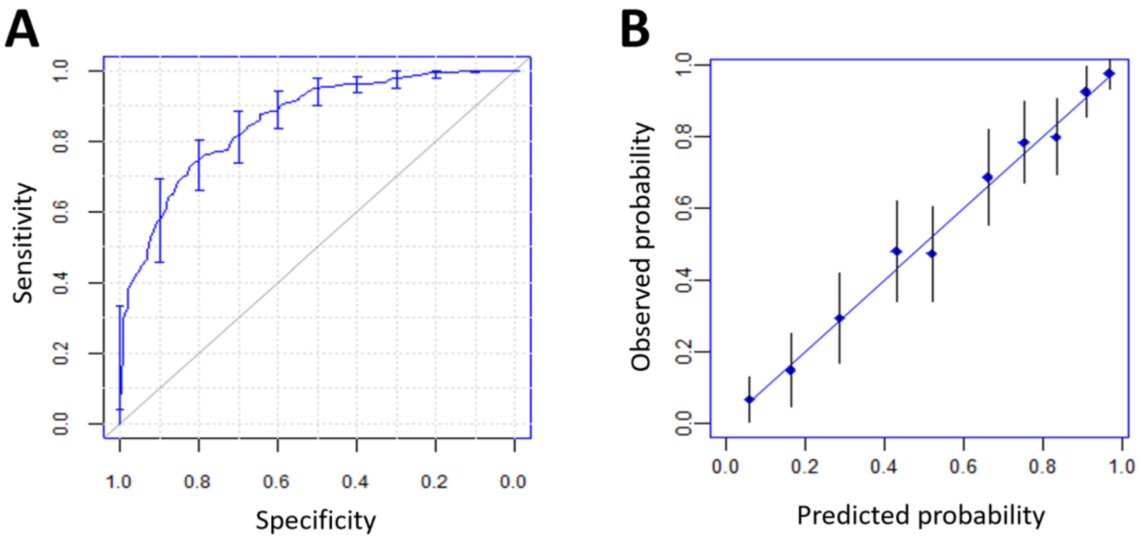

Supplementary Figure 1:

A: Discrimination of the final prognosis model, Area under the curve: AUC-ROC: 0.860, 95%CI: 0.824 to 0.8873 (DeLong estimate).

B: Calibration Assessment: Hosmer & Lemeshow test= 2.29, DF= 8, p=0.971, Fitting of Observed versus expected predictions divided in deciles: Intercept: 0.002 [-0.046 to 0.05], Slope: 1.005 [0.929 to 1.08]

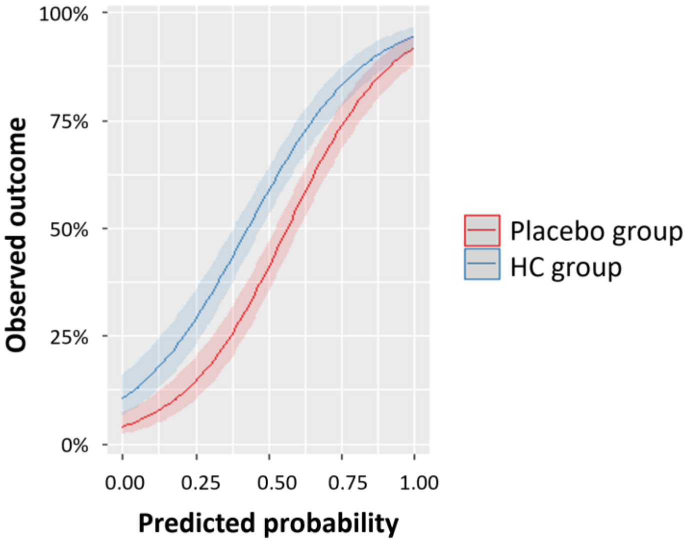

Supplementary Figure 2: Diagram of marginal estimated proportion of BPD-free survival patients (observed outcome) according to the predicting model in each treatment group.
